# *Green* Hydrogels Loaded with Extracts from *Solanaceae* for the Controlled Disinfection of Agricultural Soils

**DOI:** 10.3390/polym15224455

**Published:** 2023-11-18

**Authors:** Ilaria Clemente, Michele Baglioni, Claudia Bonechi, Flavia Bisozzi, Claudio Rossi, Gabriella Tamasi

**Affiliations:** 1 Department of Biotechnology, Chemistry and Pharmacy, University of Siena, Via A. Moro 2, 53100 Siena, Italy; michele.baglioni@unisi.it (M.B.); claudia.bonechi@unisi.it (C.B.); flavia.bisozzi@student.unisi.it (F.B.); claudio.rossi@unisi.it (C.R.); gabriella.tamasi@unisi.it (G.T.); 2 Siena Research Group-Centre for Colloid and Surface Science (CSGI), Via della Lastruccia 3, 50019 Sesto Fiorentino, Italy

**Keywords:** controlled release, biocidal action, HPLC, alginate, carboxymethyl cellulose, beads, biofumigation, pesticides

## Abstract

The UN 2030 Agenda for Sustainable Development established the goal of cutting the use of pesticides in the EU by 50% by 2030. However, a ban on pesticides could seriously affect the productivity of agriculture, resulting in severe issues due to global hunger and food deficiency. Controlled release (CR) of bioactive chemicals could play a valid alternative in this context. To this aim, two biodegradable polymers, namely sodium alginate (AL) and sodium carboxymethylcellulose (CMC), were employed to obtain crosslinked hydrogel beads for the encapsulation and CR of glycoalkaloids extracted from tomato and potato leaves to be used as biocompatible disinfectants for agricultural soils. The physico-chemical characterization of the controlled-release systems was carried out by means of Attenuated Total Reflectance–Fourier Transform Infrared (ATR-FTIR) spectroscopy, Scanning Electron Microscopy (SEM), thermogravimetry (TGA), differential scanning calorimetry (DSC) (FWI > 80%) and drying kinetics. The plant extracts and the encapsulation efficiency (~84%) were, respectively, characterized and evaluated by High-performance Liquid Chromatography–Mass Spectrometry (HPLC-MS). Finally, preliminary microbiological tests were conducted to test the efficacy of the most promising systems as biocidal formulations both in the lab and on a model soil, and interesting results were obtained in the reduction of bacterial and fungal load, which could lead to sustainable perspectives in the field.

## 1. Introduction

The widespread use of synthetic pesticides and fertilizers in agriculture since the 1950s, though granting crops protection and boosting production, generated negative consequences for the environment and risks to the health of living organisms [1,2]. Contamination and pollution of water bodies represent a serious danger both for aquatic organisms and for the human population. Moreover, pesticides and fertilizer residues in agricultural food products can be greatly harmful [3], and correlations have been confirmed between exposure to pesticides and increased incidence of several inflammatory and chronic pathologies. Therefore, regulations on their use are becoming more stringent [4,5], such that, considering their assessed impact on environmental pollution [6,7], the UN 2030 Agenda for Sustainable Development established the goal to cut the use of synthetic pesticides in the EU by 50% by 2030. However, since the world’s human population is still rapidly growing, a ban on pesticides could seriously affect the productivity of agriculture, resulting in severe issues due to global hunger and food deficiency [3]. Thus, some effective alternatives to the current use of pesticides should be carefully and promptly evaluated. Controlled release (CR) of bioactive chemicals could play a major role in this context [3,8]. CR devices should be effective, durable, highly compatible with soil and mostly biodegradable [9,10,11]. Nanoporous materials [12] are usually employed for the development of CR systems, and among them, polymeric gels likely constitute the most important and versatile matrix. In particular, physical hydrogels constituted by natural polymers and crosslinked by metal cations are deemed among the best options [13,14]. Indeed, these systems are largely employed for CR applications in pharmaceutics, cosmetics and agronomics thanks to their biodegradability, easiness of preparation, scalability and sorbent properties [15,16,17]. Specifically, in agriculture, their capacity to absorb and release water in large amounts is rather valuable [18]. Several systems were proposed over recent years by researchers based on alginate, chitosan or modified cellulose loaded with pesticides (e.g., diuron, carbofuran, atrazine, isoproturon, imidacloprid, cyromazine, chloridazon, metribuzin [3]); fertilizers (e.g., nitrogen, phosphorous and potassium—NPK [19]); plant growth inhibitors or retardants (e.g., paclabutrazol, flurpridamol, uniconazole [9]); and other active substances [8,20]. Gallagher et al. [21] proposed calcium alginate/propylene glycol alginate beads loaded with Ag+ ions with a bactericide action. Vejan et al. [22] reported on CR systems loaded with growth-promoting microorganisms Rhizobacteria. Finally, Bravo Cadena and coworkers [23] recently investigated alginate hydrogels for the CR of essential oils extracted from the bark cinnamon (Cinnamomum Zeylanicum), which are natural biocides and present a good potential as antimicrobial agents, especially if protected from fast evaporation and degradation through the inclusion in a gelled matrix. However, as just reported, the state-of-the-art in the field mainly comprises systems for the CR of synthetic pesticides. This represents a substantial improvement with respect to the uncontrolled use of free pesticides, fertilizers and other chemicals; nevertheless, it only partly meets the requirements for the concerns expressed above and rationalized in the UN 2030 Agenda for Sustainable Development. Thus, due to the necessity to develop sustainable alternatives for large-scale agriculture and the aim of reaching a zero-waste circular economy, the use of agricultural waste for added-value applications becomes extremely interesting. Food loss and waste represent a complex and challenging issue involving several variables along the food supply chain. Especially in the frame of the current global climate change, it is crucial to achieve a significant degree of waste reduction while keeping high-yield production levels [24]; in this sense, the exploitation of the valuable chemicals found in waste by-products should become a priority.

**Figure 1:**
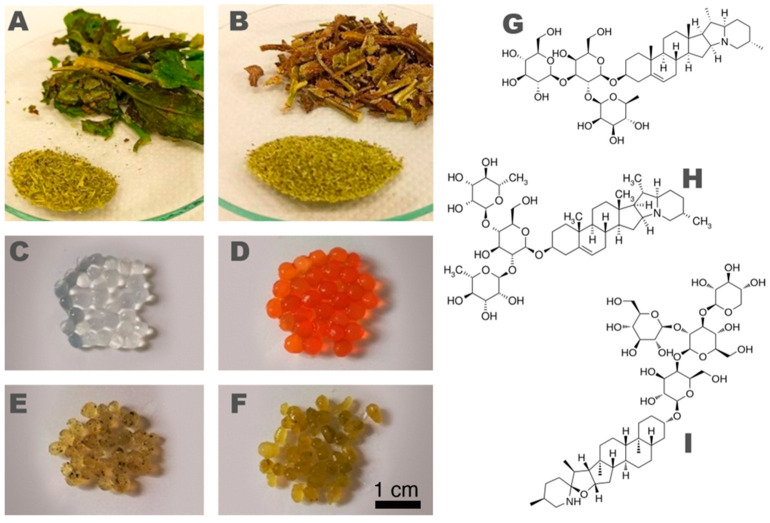
(**A**) Tomato (*S. lycopersicum*) and (**B**) potato (*S. tuberosum*) leaves, together with freeze-dried and ground leaves. (**C**) Alginate/Ca^2+^ hydrogel beads; (**D**) CMC/Fe^3+^ hydrogel beads. Alginate/Ca^2+^ hydrogel beads loaded with (**E**) tomato leaf extract and (**F**) potato leaf extract. Molecular structure of (**G**) α-solanine, (**H**) α-chaconine and (**I**) α-tomatine.

Indeed, several plant secondary metabolites, which could be extracted from agricultural wastes, are bioactive molecules with antibacterial and antifungal properties produced by plants themselves as a defense against pathogens [25]. Among crops cultivated for human nutrition, the *Solanaceae* family includes several vegetables rich in bioactive compounds, e.g., polyphenols, vitamins and carotenoids, and whose biocidal properties are due to the presence of secondary metabolites such as glycoalkaloids [26,27]. These nitrogenous compounds are derived from alkaloids, and they are formed by a hydrophobic aglycone moiety and a hydrophilic glycosidic residue. They are particularly abundant in some species of *Solanaceae*, such as tomatoes (*S. lycopersicum*) and potatoes (*S. tuberosum*). Figure 1 shows the molecular structures of some of the most relevant glycoalkaloids found in the aforementioned species.

The most abundant glycoalkaloids found in tomato are α-tomatine, which is constituted by the tomatidine aglycone and four sugars forming the glycosidic moiety, and dehydrotomatin, which is usually quantified as tomatine. Immature green tomatoes contain up to 500 mg of tomatine per Kg of fresh fruit; then the compound is rapidly degraded as tomatoes mature until the levels of about 5 mg/Kg for mature red tomatoes [28]. On the other hand, potatoes contain α-solanine and α-chaconine as the main compounds, both constituted by the aglycone solanidine but differing in the sugars of the trisaccharide residue [29].

For both plants, quantitative determinations showed that these compounds are mostly localized in the stems and leaves, and their concentration can be influenced by several factors such as geographical variety, climate, soil and, as said, the state of ripeness [29,30]. Moreover, their toxicity and efficient dosage for a biocidal effect have been investigated together with the mechanism of action, dependent on the inhibition of enzymes and the capability to disrupt the membranes in pathogens [24,31]. Considering the attractive properties of these compounds and their possible application to agricultural soils, the development of biocompatible and sustainable controlled-release systems (CRS, hereinafter) would represent a significant advancement over available methodologies based on synthetic pesticides.

To this aim, in this work, two biocompatible and biodegradable polymers, namely sodium alginate (AL) and sodium carboxymethylcellulose (CMC), were employed to obtain crosslinked hydrogel beads for the encapsulation and CR of glycoalkaloids extracted from tomato and potato leaves, to be used as biocompatible disinfectants for agricultural soils. The physico-chemical characterization of the controlled-release systems (CRS) was carried out by means of Attenuated Total Reflectance–Fourier Transform Infrared (ATR-FTIR) spectroscopy, Scanning Electron Microscopy (SEM), thermogravimetry (TGA), differential scanning calorimetry (DSC) and drying kinetics. The plant extracts and the encapsulation efficiency were, respectively, characterized and evaluated by HPLC-MS. Finally, preliminary microbiological tests were conducted to test the efficacy of the most promising systems as biocidal formulations both in the lab and on model soils.

## 2. Materials and Methods

### 2.1. Chemicals

All the solvents were purchased from Merck (Milan, Italy); food-grade commercial polymers, i.e., sodium alginate and carboxymethylcellulose, were purchased from Sigma-Aldrich (Milan, Italy) and Roeper GmbH (Hamburg, Germany), respectively. Standards for quantitative analyses (α-chaconine, α-solanine, α-solasonin, α-solamargine and α-tomatine) were purchased from Extrasynthese (Lyon, France). Plant materials (i.e., tomato and potato leaves—see Figure 1A,B) were obtained from local producers during the harvesting process in summer 2021 at the end of the productive cycle of the plants. Fresh leaves were adequately stabilized to grant the preservation of bioactive components during storage [32]. In particular, the products were washed with deionized water, dried and lyophilized (VirTis BenchTop Pro with Omnitronics, SP Scientific, Warminster, PA, USA), then freeze-dried leaves were cold-crushed in a knife mill (Pulverisette 11 Fritsch, Milan, Italy) and sieved to 500 µm particle size before storing at −20 ± 1 °C. Sabouraud dextrose agar and Sabouraud dextrose broth (supplied by Condalab, Madrid, Spain) were used as cultural media for fungi; TBX Agar (supplied by Microbiol S.r.l., Cagliari, Italy) was used as a cultural medium for *Escherichia coli*; Cetrimide agar and glycerol (supplied by Biokar Diagnostics, Allonne, France) were used for the cultural medium for *Pseudomonas aeruginosa*; and Bile Esculin Azide Agar (supplied by Biokar Diagnostics, Allonne, France) was used as a cultural medium for *Enterococcus faecalis*. Pancreatic digest, Ringer’s salts and the Plate Count Agar cultural medium were purchased from Biokar Diagnostics (Allonne, France).

### 2.2. HPLC-ESI-LTQ Quantification of Glycoalkaloids

The extraction and analytical quantification of glycoalkaloids in plant materials was carried out via HPLC-ESI-LTQ, as previously reported [32,33]. Further details are reported in Section S1.

### 2.3. Preparation and Loading of Hydrogel Microbeads

Crosslinked hydrogel beads, both “unloaded” and loaded with plant extracts, have been prepared starting from 1% *m*/*v* aqueous AL and CMC solutions. In the case of unloaded gels, polymer solutions were added dropwise into salt solutions of, respectively, 0.3 M CaCl_2_ (for alginate) and FeCl_3_ (for CMC) at RT. The forming hydrogel beads (5–8 mm diameter—see Figure 1C–F) were then magnetically stirred for 15 min and then taken out from crosslinking solutions, washed with distilled water to remove any unreacted metal ions on the surface, and stored in polyethylene containers. The preparation of the loaded hydrogel beads was carried out similarly by resuspending the dried plant extracts in an appropriate volume of acidified water (CH_3_COOH 1%, *v*/*v*) and mixing 1% *m*/*v* of the suspension with the two polymer solutions right before bead formation. Since the active glycoalkaloid content was measured to be almost equivalent and about 15–16 mg/g for both potato and tomato leaf extracts (see Section S1), the resulting theoretical concentration of the bioactive molecules in all the loaded gels prepared was approximately 1 mg/g of gel. Table 1 shows the six samples included in the characterization study.

### 2.4. Drying Kinetics and Equilibrium Water Content

The equilibrium water content (*EWC*) of the hydrogel beads was determined gravimetrically by completely drying each sample and weighing it before and afterward. All the samples were left to equilibrate beforehand by releasing excess water. *EWC* content was then calculated by using Equation (1) [34]:

(1) $$EWC=\frac{W_{wet}-W_{dry}}{W_{wet}}\times 100$$

where *W_wet_* is the weight of the swollen hydrogel, and *W_dry_* is the weight of the completely dry hydrogel. Then, the drying kinetics of the hydrogels were investigated by drying 1 g of each sample at room temperature and constant humidity and weighing them at regular intervals during the first 7 h, then again after 24 h when stable conditions were reached. The data were fitted according to the Page model [35,36] using the following equation:

(2) $$RWC=e^{-{kt}^{n}}$$

where *RWC* is the relative water content as a function of time and expresses the ratio between the amount of water in the hydrogel at time *t* and the amount of water at time *t* = 0 (the hydrogel in the equilibrium swollen state):

(3) $$RWC=\frac{W_{wet}(t)-W_{dry}}{W_{wet}-W_{dry}}$$

and where *k* is a kinetic coefficient (min^−1^), which measures how quickly the sample dries. n is an empirical constant of the model, which is introduced to modify the Newton model for drying (where *n* = 1) [35,37], i.e., the application to mass transfer of his theory on cooling of solids. In the present case, both models were tested, but the Page method yielded much better fitting curves for the experimental data. All measurements have been performed in triplicate and averaged.

### 2.5. Differential Scanning Calorimetry

Differential scanning calorimetry measurements were performed to calculate the free water index (*FWI*) of the gel systems and carried out on a DSC Q1000 (TA Instruments Leatherhead, UK), using sealed aluminum pans under an inert nitrogen atmosphere (nitrogen flow: 50.0 ± 0.5 cm^3^/min). The samples were equilibrated at −60 °C for 8 min, then heated from −60 °C up to 25 °C at 1 °C/min. The calculation of the *FWI* from fusion enthalpy values (obtained by the integration of the DTG curve peak around 0 °C) was performed according to Equation (4) [38]:

(4) $$FWI=\frac{{\Delta H}_{fus(exp)}}{WC\cdot {\Delta H}_{fus(theo)}}$$

where Δ*H_fus(exp)_* (J/g) is the experimental value of enthalpy variation relative to the melting of free water, Δ*H_fus(theo)_* (333.1 J/g) is the theoretical value of fusion enthalpy for bulk water, and *WC* is the weight of total water content in the hydrogels determined by means of the drying kinetics (see Section 2.4).

### 2.6. Thermogravimetric Analysis

Thermogravimetric measurements were performed using an SDT-Q600 (TA Instruments, New Castle, DE, USA) under nitrogen atmosphere (nitrogen flow 100 mL/min), with a heating ramp of 10 °C/min in the 30–900 °C temperature range for powder samples and empty hydrogel beads, and 30–500 °C for all other samples.

### 2.7. ATR-FTIR Spectroscopy

ATR-FTIR measurements were performed with a Nicolet IS50 FTIR spectrophotometer (Thermo Nicolet Corp., Madison, WI, USA) equipped with a single-reflection germanium ATR crystal (Pike 16154, Pike Technologies, Madison, WI, USA) and a deuterated triglycine sulfate (DTGS) detector. The spectra were acquired in the range of 4000–800 cm^−1^ at a nominal resolution of 4 cm^−1^, performing 32 scans per sample and using the spectrum of air for background correction. The frequency scale was internally calibrated with a helium–neon reference laser to an accuracy of 0.01 cm^−1^. The OMNIC software (OMNIC software system Version 9.8 Thermo Nicolet) was used for spectra acquisition and manipulation. Gelled samples were dried and equilibrated until constant weight before measurement.

### 2.8. Scanning Electron Microscopy (SEM)

Scanning Electron Microscopy micrographs were taken using a Quanta 400 SEM apparatus (FEI Company, Hillsboro, OR, USA) operating at a voltage of 20 kV. The hydrogel samples were freeze-dried to remove water and investigate them in high-vacuum conditions. Subsequently, they were placed onto stubs, with the help of a conductive bi-adhesive tape, and they were sputtered with gold to make them conductive as well. Two different magnifications were used, i.e., 80× and 600×, respectively, to take an overview of the overall features of the samples and to highlight micromorphological details both on the surface and in the inner section of the gels.

### 2.9. Encapsulation Efficiency and Release of Bioactive Compound

The encapsulation efficiency (*EE%*) in the hydrogel beads and a preliminary analysis of the release in water of bioactive molecules were estimated by means of HPLC-ESI-LTQ measurements using the same methodologies and calibration curves reported in Section S1. Since the water-soluble components of the extracts are partly released already in the crosslinker solutions during the hydrogels preparation process (see Section 2.3 for details), the encapsulation efficiency was calculated by measuring the amount of non-encapsulated glycoalkaloids in a known volume of the aforementioned solutions, and then proceeding as follows:

(5) $$EE\%=\frac{{GA}_{i}-{GA}_{m}}{{GA}_{m}}\times 100$$

where *GA_i_* is the (known) concentration of glycoalkaloids in the polymer solution that is poured dropwise in the crosslinking salt solution, and *GA_m_* is the measured concentration of glycoalkaloids in the crosslinking solution at the end of the preparation process. The release of glycoalkaloids in water was evaluated by using the same HPLC-ESI-LTQ methodology to measure the concentration of reference chemicals in water sampled from sealed vials where a weighed amount of gel beads was immersed at RT. The release solution was sampled after 4 and 21 days, and glycoalkaloids were quantified.

### 2.10. Laboratory Microbiological Tests

A series of preliminary in vitro microbiological tests was performed on the inhibitory capacity of the most promising CRS towards fungal and bacterial growth. *Aspergillus brasiliensis*, *Aspergillus fumigatus*, *Fusarium oxysporum*, *Tricophyton mentagrophytes* and *Candida albicans* were selected as the mycotic species for the tests, while *Pseudomonas aeruginosa*, *Enterococcus faecalis*, and *Escherichia coli* were selected as representative bacteria. After growing the microbial species on an appropriate culture medium (incubated at 28 °C for fungi and 36 °C for bacteria), any inhibition ring that formed around the hydrogel beads previously placed in the middle of the Petri dish was measured. All the preparations were conducted under a laminar flow hood to avoid possible contamination.

### 2.11. In Situ Tests: Treatment of a Model Soil

Finally, the disinfecting power of the better-performing CRS identified during laboratory microbiological tests was assessed in situ on a commercial model soil, which was previously characterized by measuring the main physico-chemical properties. The soil was set up inside a series of boxes for agricultural production, suitably lined with non-woven fabric to retain the soil without eliminating transpiration and humidity exchanges with the external environment. For the treatment with the CRS, the hydrogel beads were manually embedded and mixed in the ground to treat the first 5–10 cm of soil and ensure a gel/soil mass/volume ratio of about 50 g/L. Treated and untreated soils were sampled at time = 0, 10, 14, 22, 28 days, and the samples were always kept refrigerated and then analyzed within the following 24 h. The sampled soil, contained in a sterile bag, was minced and ground by hand until it was as fine as possible. Using a sterile steel spoon, 10 g of soil was taken, placed in a bottle with 90 mL of physiological solution, and stirred for 20 min.

Then, 5 mL of the solution/suspension was taken and diluted in consequential steps up to 10^−9^. Dilutions from 10^−5^ up to 10^−9^ were used for the culture examination. The Petri dishes thus prepared were incubated for 30 days at 28 ± 1 °C and held upside down (to avoid moisture condensation dripping on the agar gel). The samples used to evaluate the anaerobic bacterial load were incubated in containers sealed with plastic covers to generate an anaerobic environment. After this time, the visible colonies were counted, and results were expressed as colony-forming units per gram of soil (CFU/g).

## 3. Results and Discussion

### 3.1. Synthesis and Characterization of the CRS

The crosslinking of alginate was carried out with Ca^2+^, which is, in fact, the most commonly used ion for this purpose (though the polymer can also complex other divalent ions such as Ba^2+^ or Sr^2+^) [39]. This process involves the formation of interactions between the metal and the polar groups of the glucuronic residues that then generate the characteristic “egg-box” structure [13]. The crosslinking of CMC, on the other hand, usually involves the use of trivalent ions such as Fe^3+^, as in this work, or Al^3+^ [14]; in this case, each ion interacts with three carboxylate groups on the polymer chains. Even if alginate- and CMC-based hydrogels are physical gels, they display mechanical properties and water retention typical of chemical gels, together with a usually very high water content.

The hydrogel beads were synthesized and loaded with either potato or tomato leaf extracts as described in Section 2.3, and then characterized by means of ATR-FTIR, thermal analyses and the evaluation of drying kinetics to assess their physico-chemical properties, especially in the presence of bioactive molecules.

**Figure 2:**
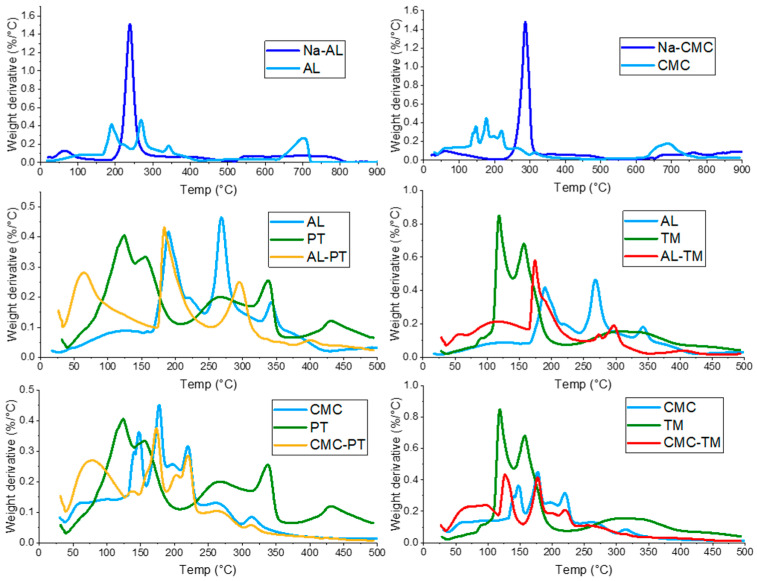
From top to bottom, DTG curves of empty AL (**left**) and CMC (**right**) hydrogels, each superposed to the corresponding pure polymer (Na-AL and Na-CMC). Curves of AL-PT (**left**) and AL-TM (**right**), CMC-PT (**left**) and CMC-TM (**right**), respectively, each superposed to curves of the relative empty sample and extracts.

Thermogravimetric analyses were carried out to evaluate the thermal behavior of the systems, particularly to evidence the effect of crosslinking on the hydrogels with respect to pure polymers and the effect of the extract encapsulation on the thermal degradation of the crosslinked polymer. The DTG curves of the two pure polymers showed three degradation stages (Figure 2, top row), i.e., the first at around 100 °C associated with the evaporation of water, the second between 200 and 300 °C due to degradation of polymer chains, and the last in the 550–800 °C range, where decomposition of Na_2_CO_3_ in Na_2_O and CO_2_ occurs [40,41]. Pure CMC seemed to be more thermally stable since the degradation peak was shifted at a slightly higher temperature (250–300 °C) (Figure 2, right, top row). Crosslinked “unloaded” hydrogels (AL and CMC) showed weight loss due to water evaporation up to 120 °C, even though the loss of coordination water was likely superposed to the degradation peak of the chains. The crosslinking effect could be noticed particularly in the 150–300 °C range, where degradation of polymer chains occurs. Indeed, the curves of empty AL showed two degradation peaks in the 160–350 °C range, the first event likely being relative to weakly reticulated residues at lower temperatures with respect to pure alginate, whereas the second degradation shifted at higher temperatures was associated with the “egg-box” crosslinked residues. Contrarily, empty CMC decomposition occurred in several steps in the 140–220 °C range at lower temperatures with respect to the pyrolysis peak observed for the pure polymer DTG plot. It can be hypothesized that the presence of Fe^3+^ ions in some way catalyzed and thus favored the thermal degradation of polymeric chains. For both hydrogels, a third event was evidenced in the 600–800 °C range due to the decomposition of inorganic compounds, e.g., calcium carbonate for the AL sample. At the end of the heating cycle, the inorganic residue stable above 900 °C was estimated at 25% for both samples.

The DTG curves of samples (Figure 2, second and third row) loaded with the extracts showed the most relevant thermal events in the 120–200 °C range, corresponding to the loss of volatile molecules, whereas the decomposition of macromolecules and depolymerization of carbohydrates to sugars occurred in the 200–500 °C range. Indeed, glycoalkaloids can be considered fairly thermostable molecules, so their degradation starts above 350–380 °C. The thermal ramp for loaded hydrogels was set up to 500 °C to assess the influence of encapsulated extracts on the thermal degradation of crosslinked polymers. Though it was not possible to evidence substantial events relative to the extracts due to the much higher polymer content, it could be noticed a shift to higher temperatures of the pyrolysis peaks likely ascribable to the thermal degradation of alginate for AL-based hydrogels (Figure 2). Specifically, the second degradation peak of the polymer chains resulted in a shift of about 30 °C, suggesting that the interactions of the polymer with the extract induced higher stability and, thus, a slight increment of the degradation temperature. On the other hand, for CMC-loaded hydrogels, no noticeable influence of extract encapsulation on thermal behavior was evidenced.

ATR-FTIR measurements qualitatively confirmed the presence of encapsulated compounds after preparation. Band assignments for “unloaded” samples (AL and CMC) compared with pure polymer powders are reported in Table 2. The effect of crosslinking on hydrogels with respect to powders could be noticed particularly in the spectral range 1700–900 cm^−1^, which can be subdivided in the 1700–1400 cm^−1^ range where absorbances of carbonyl groups are found, and the fingerprint range at 1400–900 cm^−1^. Shifts relative to the carboxylate anion stretching, both asymmetric (1650–1550 cm^−1^) and symmetric (1400 cm^−1^) at bigger wavenumbers were evidenced [42,43]. Similarly, in the spectrum registered on CMC, “unloaded” hydrogel beads, the carbonyl asymmetric stretching was shifted at higher wavenumbers.

**Figure 3:**
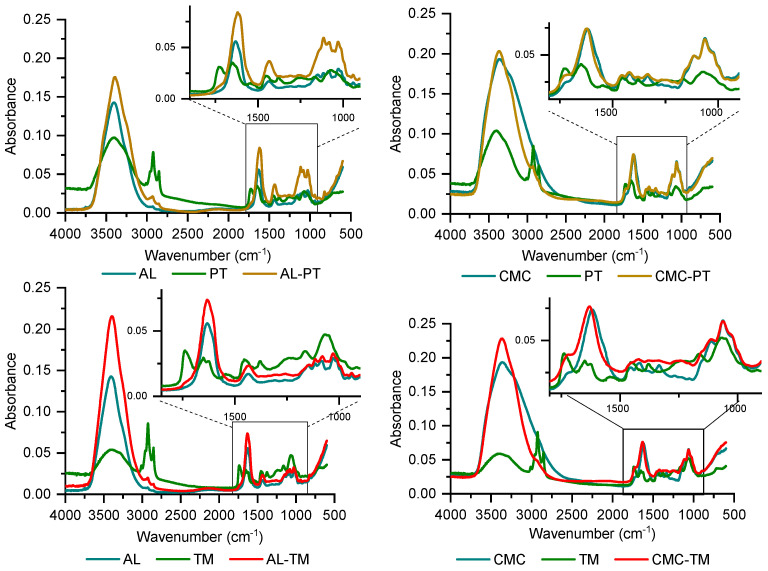
FTIR spectra of AL−PT (**top**, **left column**) and AL−TM (**bottom**), each superposed to empty AL and the corresponding extract. FTIR spectra of CMC−PT (**top**, **right column**) and CMC−TM (**bottom**), each superposed to empty CMC and the corresponding extract.

The analysis performed on loaded hydrogels confirmed the presence of encapsulated compounds. Particularly, Figure 3 (left column) shows the spectra of loaded alginate hydrogels, each superimposed to the spectra of empty AL and the corresponding extract, i.e., from potato or tomato leaves. Peak assignments for extracts are reported in Table 3 [30,44]. It can be noticed that the spectra relative to extracts and loaded hydrogels are rather similar due to the presence of mostly the same chemical groups (Table 2 and Table 3). However, it was possible to evidence the presence of encapsulated extracts from the absorbance at around 1730 cm^−1^, where a shoulder attributed to the ester carbonyl stretch could be seen in the spectra of both extracts and loaded hydrogels but not in the empty AL curve (Figure 3, left). Moreover, the higher absorbance intensity of loaded hydrogels in the fingerprint 1300–1000 cm^−1^ range was likely due to the contribution of the extracts as well. For CMC-loaded hydrogels, the contribution of the encapsulated compounds could be evidenced by the enhanced intensity of the shoulder at 1730 cm^−1^ with respect to empty CMC (Figure 3, right column) and from the shift of the carbonyl asymmetric stretch at around 1629 cm^−1^. Convolution between polymer and extract bands could be seen in the range of 1500–1000 cm^−1^, where the spectra of loaded hydrogels showed an enlarged band.

**Figure 4:**
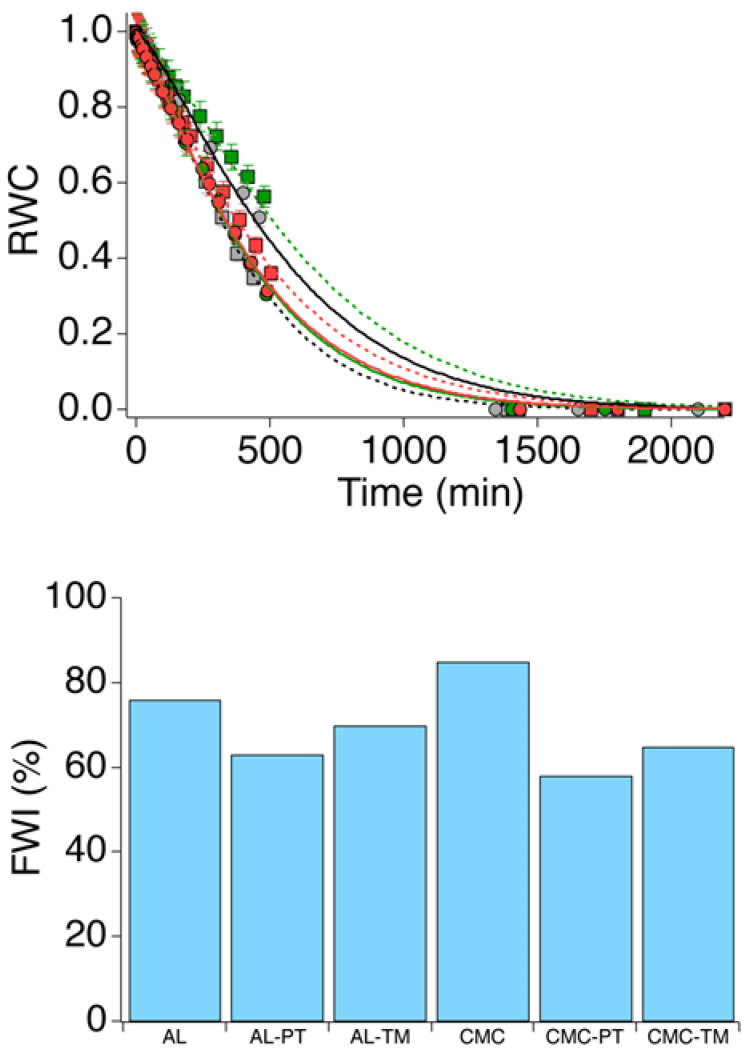
(**Top**) Drying kinetics experiments. AL, grey squares; AL-PT, green squares; AL-TM, red squares; CMC, grey circles; CMC-PT, green circles; CMC-TM, red circles. Solid and dashed lines represent fitting curves obtained using the Page model. (**Bottom**) Histogram reporting the FWI for the six investigated samples.

Then, drying kinetics were carried out to assess the hydrophilicity and water retention capacity of the loaded and “unloaded” hydrogels. The equilibrium water content (*EWC*) was first obtained, as described in Section 2.4. All the hydrogels showed a very high average *EWC* of 93 ± 2%. On the other hand, however, the drying kinetics followed different trends for the six samples. The top graph of Figure 4 shows the experimental data fitted according to the Page model, as described in detail in Section 2.4, while Table S3 reports the complete fitting parameters, *k* and *n*, being, respectively, the drying rate and the Page exponent. Interestingly, *n* is fairly constant among investigated samples and approximately equal to 1.2–1.3, indicating a slight deviation from the Newton model on drying kinetics. That said, the trend of *k* is somewhat noteworthy. Particularly, the “unloaded” AL hydrogel showed a faster dehydration with respect to empty CMC (*k_AL_* is about 40% larger than *k_CMC_*), whereas for loaded samples, the presence of encapsulated extracts increased the drying speed for CMC-based gels, and, contrarily, decreased it for AL-based samples.

To further deepen the picture, DSC measurements were performed to estimate the FWI of the hydrogels, both in the absence and in the presence of leaf extracts. According to the interactions between water and polymer chains, the thermodynamic properties of water, such as the freezing temperature, can vary. Specifically, free water not interacting with the polymer shows the same behavior as pure water, thus freezing at 0 °C; freezable bound water interacts weakly, thus freezing at temperatures lower than 0 °C; and non-freezable water bound by strong interactions does not freeze at temperatures higher than −70 °C. The bottom graph of Figure 4 shows the *FWI* values for the investigated samples.

From the obtained values, it was noted that the lower *FWI* in the AL hydrogel with respect to CMC indicates a slightly higher capability of the alginate chains to interact with water through OH and COO- groups [45]. Indeed, in CMC, the presence of Fe^3+^ ions coordinating carboxylate groups might allow fewer interactions with water than Ca^2+^ in alginate samples. The encapsulation of the extracts in the hydrogels induced a decrease in the *FWI* for all samples, particularly in CMC hydrogels. This can be likely attributed to the effect of the complex mixture of biomolecules included in the leaf extracts, which increases the amount and the “strength” of the interactions with water molecules (a relevant contribution is surely due to the sugar portions of glycoalkaloid molecules, whose OH groups can efficiently establish hydrogen bonds). Contrary to “unloaded” systems, loaded AL samples showed higher *FWI* than loaded CMC samples. Nevertheless, all samples showed high free water content, as expected for this type of system.

**Figure 5:**
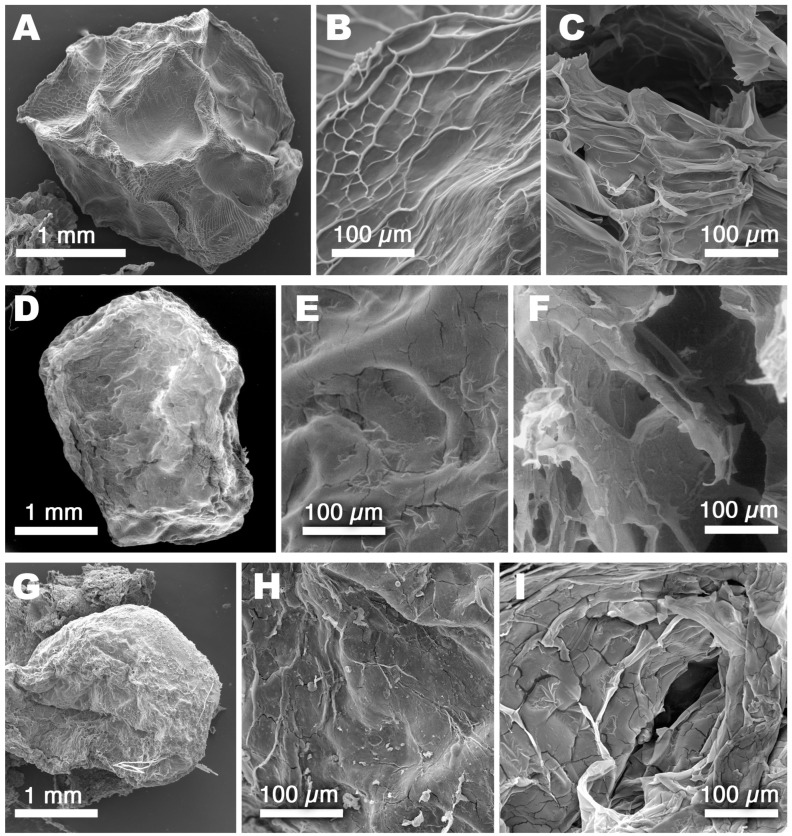
SEM micrographs taken on freeze-dried hydrogel beads. (**A**) AL gel bead; (**B**) AL bead surface; (**C**) AL inner microstructure (exposed section); (**D**) CMC gel bead; (**E**) CMC bead surface; (**F**) CMC inner microstructure (exposed section); (**G**) AL−PT gel bead; (**H**) AL−PT bead surface; (**I**) AL−PT inner microstructure (exposed section). The micrographs were chosen as a representative selection of all samples, which all presented similar features and no significant differences.

Finally, the microstructure of the hydrogel beads was investigated by means of SEM analyses. Figure 5 reports a selection of significant micrographs taken on, respectively, AL “unloaded” hydrogel beads (Figure 5A–C), CMC “unloaded” hydrogel beads (Figure 5D–F), and AL−PT, i.e., Alginate/Ca^2+^-based hydrogels loaded with extracts from potato leaves (Figure 5G–I). Overall, the micrographs report the typical features of such systems in good agreement with other studies [46]. It is worth noting, though, that the freeze-drying process needed to remove water for the analysis in high-vacuum conditions produced severe mechanical stress on such highly hydrated hydrogels. This can be clearly observed in Figure 5A,D,G where gel beads appear shrunken and deformed if compared to the smooth, globularly shaped beads reported in Figure 1. The close-up of the surface micromorphology of all the investigated samples (Figure 5B,E,H) evidenced wrinkles and cracks, which can be likely interpreted as artifacts due to the beads’ shrinkage during freeze-drying. Most interestingly, the observation of exposed gel sections (Figure 5C,F,I) allowed an overview of the inner micromorphology of the samples, highlighting the presence of large pores (tenths to hundreds of µm)—which appear collapsed, as a result of freeze-drying—that are coherent with hydrogels having an *EWC* of 93%. Even if the comparison between different samples is tricky due to the samples’ deformation, the loading of the extracts does not seem to affect the micromorphology of the hydrogels and can be only spotted due to the presence of some small clumps and particles (look at Figure 5H,I), of about 10–20 µm, adhered to gel walls. These cannot be observed on “unloaded” samples and were identified as small clusters of water-insoluble matter coming from the vegetal extracts.

The picture emerging from the physico-chemical characterization of the investigated samples shows a quite complex interaction between the polymeric backbones of the hydrogels and the multi-component extracts from potato and tomato leaves. Overall, both AL- and CMC-based hydrogels show promising properties in view of an application as CRS in soils featuring high *EWC*s and *FWI*s.

### 3.2. Assessment of the Efficiency of the Selected CRS

According to their physico-chemical characterization, all the considered hydrogels, namely AL−PT, AL−TM, CMC−PT and CMC−TM, can be potentially equally suitable for the application of CRS for the disinfection of soils. Thus, it was decided to focus on just one system to measure the *EE%*, the release of glycoalkaloids in water, the biocidal effect on microorganisms grown in Petri dishes, and finally, the biocidal effectiveness on a commercial model soil. To select the CRS, we then focused on the activities of the bioactive compounds in the extracts.

It is generally known that glycoalkaloids have some biocidal activity on insects (such as *Heliothis viresecens*, *Manduca sexta*, *Spodoptera frugiperda*, *Podisus maculiventris*, and others [24]) since they—similar to most pesticides—are also inhibitors of the acetylcholinesterase and butyrylcholinesterase enzymes, which catalyze the hydrolysis of acetylcholine at the synapse in the nervous systems of these animals [24]. They are also inhibitory towards several fungi (e.g., *Alternaria brassicicola*, *Phoma medicaginis*, *Ascobolus crenatulus*, *Rhizoctonia solani* [24], *Fusarium caeruleum* [47]), and they were reported to reduce the spore germination of different species [48]. The mechanism proposed for their biocidal activity on fungi involves the insertion of the aglycone portion of glycoalkaloids in the phospholipid bilayer structure of cell membranes in correspondence with the ergosterol location. Ergosterol is a sterol and one of the main components in the cell membranes of fungi and protozoa, where it has the same function as cholesterol in more complex animal cells. Once the aglycone/sterol interaction is established, a second interaction between the sugar moieties of glycoalkaloids is likely to occur, which creates a rigid sterol–glycoalkaloid matrix. This complex perturbs the membrane functions and finally causes the lysis of the cell [24,28,49]. Several factors can affect this mechanism, but pH certainly is one of the most relevant. It was shown that the biocidal activity of glycoalkaloids is inhibited at acid pH, mainly due to the protonation of the N atom in the steroidal portion of the molecule. That results in a positive charge in the steroidal portion of glycoalkaloids, which become less lipophilic and thus less prone to interact with the sterols of cell membranes of fungi. This is why both α-tomatine and α-solanine tend to be inactive in the protonated form at pH 4 and active in the unprotonated form at pH 7 and above [28,47,48,50]. If we compare the biocidal activity of α-tomatine and α-solanine, it is generally accepted that, though greatly depending on pH, the LD50 of α-tomatine is lower than that of α-solanine [45,49], i.e., α-tomatine tends to be more toxic to fungi.

Thus, according to the data reported in the literature, it was decided to focus on tomato leaf extracts rich in tomatine. Furthermore, in order to maximize the biocidal action in view of applicative tests, the concentration of the extract in the polymer solution (prior to crosslinking with metal ions) was increased 10 times so as to reach a theoretical concentration of the bioactive molecules in the final gel of approximately 10 mg/g of gel. However, this was possible only for CMC-based hydrogels since the alginate solution began to gel before it was added dropwise to the salt solution, most likely due to the presence of a significant amount of Ca^2+^ ions in the tomato leaf extract. As a result, the CMC-TM formulation was selected as the CRS to be assessed in the applicative experiments.

Firstly, the encapsulation efficiency (*EE%*) of the hydrogel was measured. The release of encapsulated compounds from gel beads during the stirring step is due to the concentration gradient between the hydrogel itself and the crosslinker solution, and it is widely reported in the literature. Nevertheless, the measured *EE%* was high (84 ± 5%), likely due to the poor water solubility of glycoalkaloids and their affinity for the polymers. Probably for the same reason, the release of glycoalkaloids in water measured after 4 and 21 days was just 2.8 ± 0.1%, which is poorly representative of what would happen in the soil where other mechanisms occur, possibly involving the erosion and final complete disintegration of the hydrogel medium due to physical, chemical, and biological degradation of the polymeric matrix [9]. However, the result obtained can be seen as promising since it also suits the context of controlled release, where the gradual and slow migration of the bioactive compounds would be optimal.

Then, the biocidal activity of CMC−TM was tested in vitro by performing microbiological tests on Petri dishes, as reported in Section 2.10, on a selection of bacteria and fungi species. Table 2 reports the inhibition ring measured for each microorganism. It is worth noting that the inhibition ring measured for the reference “unloaded” CMC hydrogel was 0 mm for all the tested microbial species, showing that the efficiency observed for the CMC-TM system should be ascribed to the bioactive compounds in the extract from tomato leaves. While a good biocidal activity towards fungi was expected in view of the aforementioned literature data, some effectiveness to bacteria was also observed. Moreover, the results were quite comparable to data reported in the literature by Gallagher et al. concerning the biocidal action of similar hydrogels loaded with Ag^+^ ions on these strains [21]. As a useful term of comparison, the growth inhibition effect of a series of standard synthetic pesticides on several different bacteria and fungi is reported in Table S4 [51], while the aforementioned results of Gallagher et al. [21] are reported in Table S5. It is worth noting that, even if concentrations employed in these experiments are crucial (thus, the comparison should be handled with care), among standard pesticides tested, only Carbendazim (chemically, a Methyl (1H-1,3-benzimidazol-2-yl) carbamate whose use was severely reduced due to its toxicity) produced similar appreciable results on some fungi strains, i.e., *Fusarium oxysporum* and *Trichoderma harzianum*. On the other hand, the use of Ag+ ions loaded into alginate-based gels provided appreciable results on several bacteria species, e.g., *Staphylococcus aureus* and *Escherichia coli*, but the repeated introduction of Ag+ ions in agricultural soils certainly poses some ecological concerns.

After these promising preliminary tests, the CMC−TM CRS was finally tested in an experiment simulating a real application on an agricultural soil. A commercial soil was selected and characterized, measuring some standard physico-chemical parameters (see Table S4). The pH of the soil was 7.5, which, as said, is suitable to observe some biocidal activity of glycoalkaloids. The experiment was conducted as detailed in Section 2.11, and Figure 5 reports the final results obtained by plotting the colony-forming units (CFU) per g of culture soil as a function of sampling time (days).

**Figure 6:**
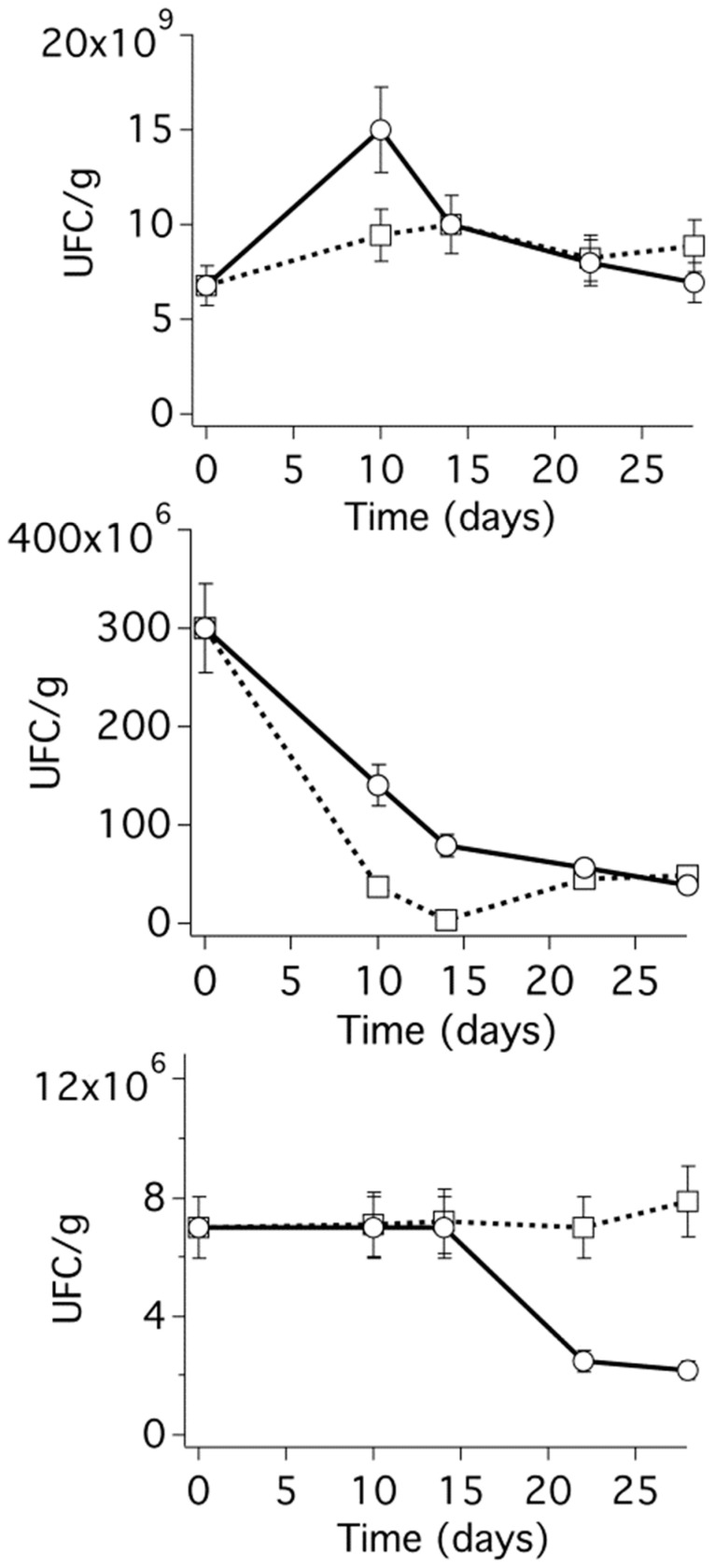
(**Top**) Aerobic bacterial load. (**Middle**) Anaerobic bacterial load. (**Bottom**) Fungal load. The experimental of both the treated (circles) and untreated reference (squares) soils are reported together with, respectively, solid and dashed lines to be used as eye guides.

The top graph of Figure 6 shows the evolution of the total aerobic bacterial load in the soil during the 28 days of the experiment, and, in part, it could be expected that in light of the limited effectiveness of glycoalkaloids on bacteria, no significant effect could be evidenced. This might seem to contradict what is reported in Table 4, where some slight efficacy was also observed on three bacterial strains, but in the experiment performed in the actual soil, the bacterial species were not identified, for the attention was preliminarily focused on the reduction of the overall microbial load. On the other hand, the middle graph of Figure 6 shows the trend of the total anaerobic bacterial load in the soil during the experiment period. In this case, a significant decrease in the microbial load was observed both for the treated and the reference soil, most likely as the result of the scarce resistance of anaerobic bacteria in the upper 10 cm of a soil exposed to air. Finally, the bottom graph in Figure 6 shows a significant reduction in the fungal load with respect to the untreated reference soil, starting two weeks after the treatment of the soil with CMC-TM hydrogel beads. This is a very promising result since it demonstrates the potential of this methodology both in terms of biocidal activity to fungi and in terms of controlled release.

## 4. Conclusions

In the present paper, two biocompatible and biodegradable polymers, sodium alginate (AL) and sodium carboxymethylcellulose (CMC), were selected to synthesize crosslinked hydrogel beads for the encapsulation and CR of glycoalkaloids extracted from tomato and potato leaves to be used as a “green” disinfectants for agricultural soils. DTG measurements showed a 30 °C shift to higher temperatures of the pyrolysis peaks due to the thermal degradation of alginate for AL-based hydrogels, which could account for the interactions established between the polymer and the components of the extract. The ATR-FTIR analysis performed on loaded hydrogels confirmed the presence of encapsulated compounds. DSC and drying kinetics showed that all investigated gels, independently of the presence of extracts, have the same *EWC*. The *FWI* of the AL “unloaded” hydrogel was found to be lower than the CMC one, but the former dried faster. In both cases, the extracts decreased the *FWI*, but for AL-based systems, the drying rate increased, while for CMC-based hydrogels, it decreased upon loading. This complex behavior is not easily explainable, considering the concurrent and synergistic interactions between the two polymers and the multi-component vegetal extracts, which, besides glycoalkaloids, also include a range of other molecules. However, according to their physico-chemical characterization, all the considered hydrogels, namely AL−PT, AL−TM, CMC−PT and CMC−TM, resulted in being equally suitable for the application of CRS for the disinfection of soils. We decided then to focus on CMC−TM to measure the *EE%*, the release of glycoalkaloids in water, the biocidal effect on microorganisms grown in Petri dishes, and finally, the biocidal effectiveness on a commercial model soil. Firstly, the encapsulation efficiency (*EE%*) of the hydrogel was high (84 ± 5%), likely due to the poor water solubility of glycoalkaloids and their affinity for the polymers. Probably for the same reason, the release of glycoalkaloids in water measured after 4 and 21 days was only 2.8 ± 0.1%. Then, the biocidal activity of the selected CRS was tested in vitro, and while a good biocidal activity towards fungi was expected, some effectiveness on bacteria was also observed. CMC−TM was finally tested in an experiment simulating a real application on agricultural soil, where it produced a significant reduction in the fungal load with respect to the untreated reference soil. Overall, we showed the potential of a class of biocompatible and environmentally friendly products that exploit by-products of agro-industrial productions, such as tomato and potato leaves, in the context of circular economy and sustainability, which proved to be suitable as disinfection treatments for agricultural soils. The controlled release of glycoalkaloids widens the range of methodologies that can be used mainly to reduce the presence of fungi species in the soil, several of which are potentially harmful to agricultural production.

## Figures and Tables

**Table 1:** Sample list showing the composition and nomenclature used for each system.

Samples	Acronyms
Alginate-Ca^2+^	AL
Alginate-Ca^2+^+ potato leaf extract	AL-PT
Alginate-Ca^2+^+ tomato leaf extract	AL-TM
CMC-Fe^3+^	CMC
CMC-Fe^3+^+ potato leaf extract	CMC-PT
CMC-Fe^3+^+ tomato leaf extract	CMC-TM

**Table 2:** Spectral assignment for empty hydrogels (AL and CMC) compared to pure polymers (poly-AL and poly-CMC).

Assignment	Wavenumber (cm^−1^)			
	poly-AL	AL	poly-CMC	CMC
Stretching –OH	3650–3000	3650–3000	3625–3000	3700–2650
Stretching –CH	2943	2930	2898	-
Stretching –COO– (asymm.)	1605	1627	1596	1620
Stretching –COO– (symm.)	1410	1434	1417	1417
Stretching –C–O–C–	1067	1086	1058	1062

**Table 3:** Spectral assignment for PT and PM.

Assignment	Wavenumber (cm^−1^)	
	PT	TM
Stretching –OH/–NH	3407	3419
Stretching –CH_3_/–CH_2_	3010–2850	3010–2850
Stretching C=O esters	1729	1738
Stretching C=O amides	1650	1650
Stretching C=C–C aromatic	1627	1626
	1530	1530
Bending –OH	1240	1230
Stretching C–O	1169	1164
Stretching C–O–C glycosidic	1070	1069

**Table 4:** Results of the microbiological tests on Petri dishes for the biocidal activity of the CMC−TM CRS. The inhibition ring for each microbial species is reported in mm.

	Microorganism	CMC	CMC−TM
Bacteria	*Escherichia coli*	0 mm	8 mm
	*Pseudomonas aeruginosa*	0 mm	22 mm
	*Enterococcus faecalis*	0 mm	13 mm
Fungi	*Aspergillus brasiliensis*	0 mm	20 mm
	*Aspergillus fumigatus*	0 mm	10 mm
	*Fusarium oxysporum*	0 mm	15 mm

## Data Availability

Data are contained within the article and Supplementary Materials.

## Supplementary Materials

The following supporting information can be downloaded at https://www.mdpi.com/article/10.3390/polym15224455/s1. Table S1: Analyte list, retention time (tR), mass and *m/z* values of identified glycoalkaloids in tomato and potato leaves extracts; Table S2: Calibration curves used for glycoalkaloids quantitative determination; Table S3: Fitting coefficients extracted from the drying curves; Table S4: Reported values in literature of standard pesticides growth inhibition effect; Table S5: Reported values in literature of growth inhibition of Calcium/alginate hydrogels unloaded or loaded with silver (I) ions; Table S6: Physico-chemical parameters measured on the commercial soil selected for the experiments on the biocidal activity of CMC-TM.
