# Supplementary material for: Green Hydrogels Loaded with Extracts from Solanaceae for the Controlled Disinfection of Agricultural Soils
Source: Polymers (Basel). 2023 Nov 18;15(22):4455. doi: 10.3390/polym15224455 (PMC10674755; doi:10.3390/polym15224455)
Supplement: Supplementary file 1 [file polymers-15-04455-s001.zip › polymers-2613842-supplementary.pdf]

SUPPLEMENTARY INFORMATION
FOR

Green Hydrogels Loaded with Extracts from *Solanaceae* for the Controlled Disinfection of Agricultural Soils

Ilaria Clemente [1,2,*], Michele Baglioni [1,2], Claudia Bonechi [1,2], Flavia Bisozzi [1,2], Claudio Rossi [1,2] and Gabriella Tamasi [1,2]

[1] Department of Biotechnology, Chemistry and Pharmacy, University of Siena, Via A. Moro 2, 53100 Siena, Italy; michele.baglioni@unisi.it (M.B.); claudia.bonechi@unisi.it (C.B.); flavia.bisozzi@student.unisi.it (F.B.); claudio.rossi@unisi.it (C.R.); gabriella.tamasi@unisi.it (G.T.)
[2] Siena Research Group-Centre for Colloid and Surface Science (CSGI), Via della Lastruccia 3, 50019 Sesto Fiorentino, Italy
* Correspondence: ilaria.clemente2@unisi.it

Keywords: controlled release; biocidal action; HPLC; alginate; carboxymethyl cellulose; beads; biofumigation; pesticides

---

S1. HPLC-ESI-LTQ Quantification of Glycoalkaloids

Briefly, lyophilized samples were extracted in triplicate with a hydroalcoholic mixture of water and ethanol (EtOH) (70:30, v/v), acidified with acetic acid 1%; the three-cycles extraction on the solid phase was performed in an ultrasonic bath (10 min, 20 ± 1 ◦C; nominal power 120 W; ultrasound frequency 35 kHz; Branson Ultrasonics Corporation, Danbury, CT, USA). The suspension was then centrifuged (Thermo Electron Corporation PK 110 centrifuge), and the supernatant was carefully separated from the solid residue and transferred into a polypropylene tube. The extracts were dried under nitrogen flow, lyophilized and finally reconstituted in methanol (MeOH)/H_2O (60:40, v/v). The analytical quantification of glycoalkaloids was carried out with a HPLC instrument (Thermo Fisher Scientific UltiMate3000) coupled to a linear ion trap mass spectrometer (Thermo Fisher Scientific LTQ XL), equipped with an electrospray ion source (ESI). The spectra were acquired and processed using Xcalibur software (Thermo Fisher Scientific, Waltham, MA, USA). A Phenomenex Kinetex C18 Polar column (150 x 2.1 mm, 5 µm, 100 Å) was used, equipped with a Phenomenex C18 Polar pre-column (4 x 2.0 mm) thermostated at 35 °C. The separation was performed with eluents (A) H_2O and (B) acetonitrile, both acidified with formic acid 0.1% (v/v) at 0.4 mL/min flow rate, using the following gradients: from 0.0 to 1.0 min 20% B (isocratic), from 1.0 to 23.0 min 20-50% B (linear), from 23.0 to 25.0 min 50-95% B (linear), from 25.0 to 29.0 min 95% B (isocratic), from 29.0 to 30.0 min 95-20% B (linear), from 30.0 to 40.0 min 20% B (isocratic). The injection volume was 3 µL. The ESI-MS conditions (spray voltage 5000 V; sheath gas and auxiliary gas pressure of 35 and 25 arbitrary units respectively; capillary temperature 200 °C) were optimized through the direct injection of glycoalkaloids standard solution in MeOH (1 mg/L) in positive ionization mode in a 25% B mobile phase flow. The single ion monitoring (SIM) was used for quantitative targeted determination with multi-analyte (α-chaconine, α-solanine, α-solasonine, robeneoside-A, robeneoside-B, α-solamargine, α-tomatine, dehydrotomatin and β1-tomatine, with peimine as internal standard) method. All molecules were quantified using the external calibration

method with internal standard. The calibration curves of the analytes were acquired in triplicate and obtained by plotting the area ratio of the analytes normalized by the internal standard against the analyte concentration in the linearity ranges. The LOQ and LOD were 0.025 ppm and 0.0083 ppm respectively for potato glycoalkaloids, and 0.05 ppm and 0.017 ppm respectively for tomato ones. Dehydrotomatin and β1-tomatine were expressed as α-tomatine mg-equivalents, whereas robeneoside-A, robeneoside-B e α-solamargine as α-solasonine equivalents. The resuspended extracts were filtered through a 0.2 μm PTFE syringe filter (Whatman) before injection, and analyzed. Samples and standards were injected and analyzed in triplicate.

The preliminary identification of the main bioactive compounds in the two extracts were carried out through fragmentation analysis and data dependent acquisition approach, by comparing the MS^n spectra produced from the sequentially fragmented compounds in the ion trap with the spectra of characteristic fragments obtained for standards and/or reported in spectral databases (MassBank, mzcloud, HMDB).[1] Fragmentation analysis was performed by collecting MS scans above a threshold level and fragmenting the most abundant ion through CID (collision induced dissociation), using He as the collision gas, and thus generating a MS^2 spectra. The LC-MS procedure allowed to quantify the concentration of glycoalkaloids per gram of dry leaves, expressed respectively as α-tomatine equivalents for tomato extract, and α-chaconine and α-solanine equivalents for potato extract. The resulting glycoalkaloids contents were 15.4 ± 0.2 mg/g and 16.4 ± 0.1 mg/g for potato and tomato leaves respectively. Parameters and corresponding calibration curves are reported in **Tables S1** and **S2**.

Table S1. Analyte list, retention time (t_R), mass and m/z values of identified glycoalkaloids in tomato and potato leaves extracts.

Analyte	t_R (min)	Mass (u.m.a.)	$[M+H]^+$
peimine (IS)	4.77	431.7	432.5
robenoside-B	7.73	900.1	900.7
robenoside-A	8.65	883.7	884.1
α-solasonine	12.04	884.1	884.7
α-solanine	12.61	868.1	868.8
dehydrotomatin	13.01	1032.1	1032.7
α-chaconine	13.37	852.1	852.7
α-solamargine	13.84	868.1	868.7
β1-tomatine	14.07	902.1	902.7
α-tomatine	14.87	1034.2	1034.8

Table S2. Calibration curves used for glycoalkaloids quantitative determination.

Analyte	Curve equation	R^2	Linearity range
α-chaconine	Y = 3.59442X - 0.00281785	0.9999	0.025 – 2.5 mg/L
α-solanine	Y = 2.93831X - 0.00177654	0.9999	0.025 – 2.5 mg/L
α-solasonin	Y = 1.694X - 0.00143757	0.9999	0.025 – 10 mg/L
α-tomatine	Y = 0.34527X + 0.0317194	0.9981	0.05 – 10 mg/L

S2. Drying Kinetics Fitting

Table S3. Fitting coefficients extracted from the drying curves.

Sample	*k* (min^{-1})	*n*
AL	0.00234 ± 0.00003	1.28 ± 0.03
AL-PT	0.00149 ± 0.00005	1.35 ± 0.06
AL-TM	0.00195 ± 0.00003	1.20 ± 0.03
CMC	0.00170 ± 0.00006	1.30 ± 0.06
CMC-PT	0.00222 ± 0.00003	1.22 ± 0.03
CMC-TM	0.00220 ± 0.00003	1.19 ± 0.02

S3. Literature reference values on the biocidal activity of standard pesticides and silver-ion loaded similar gels

Table S4. Reported values in literature of standard pesticides growth inhibition effect.[6]

	Microorganism	Acetochlor	Carbendazim	Chlorpyrifos	EPTC	Simazine
Bacteria	*Bacillus subtilis*	0 mm	0 mm	0 mm	5 mm	0 mm
	Mycobacterium phlei	1 mm	0 mm	0 mm	4.6 mm	1.3 mm
	Pseudomonas Fluorescens	2.3 mm	0 mm	0 mm	7 mm	0 mm
Fungi	*Fusarium Oxysporum*	0 mm	10 mm	0 mm	0 mm	0 mm
	Penicillium expansum	0 mm	0 mm	0 mm	6.3 mm	0 mm
	Trichoderma Harzianum	0 mm	24 mm	0 mm	0 mm	0 mm

Table S5. Reported values in literature of growth inhibition of Calcium/alginate hydrogels unloaded or loaded with silver (I) ions.[7]

Polymeric material	C. Albicans	MRSA	S. aureus	E. coli	P. aeruginosa 10145	P. aeruginosa 27853
ALG (no Ag+)	0 mm^2	0 mm^2	0 mm^2	0 mm^2	0 mm^2	0 mm^2
ALG/Ag + (0.01)	Direct inhibition	23 mm^2	19 mm^2	31 mm^2	17 mm^2	23 mm^2
ALG/Ag+ (0.1)	83 mm^2	99 mm^2	21 mm^2	39 mm^2	21 mm^2	30 mm^2

S4. Physico-chemical parameters of the selected commercial soil

Table S6. Physico-chemical parameters measured on the commercial soil selected for the experiments on the biocidal activity of CMC-TM.

Parameter	Measure
pH	7.5
Porosity % (v/v)	89
Total organic carbon	30%
Organic matter	52%
Total nitrogen	< 2%
Humic acids (% s.s.)	62
Fulvic acids (% s.s.)	49

References

1. Cangeloni, L.; Bonechi, C.; Leone, G.; Consumi, M.; Andreassi, M.; Magnani, A.; Rossi, C.; Tamasi, G. Characterization of Extracts of Coffee Leaves (Coffea Arabica L.) by Spectroscopic and Chromatographic/Spectrometric Techniques. *Foods* 2022, *11*.

2. Papageorgiou, S.K.; Kouvelos, E.P.; Favvas, E.P.; Sapalidis, A.A.; Romanos, G.E.; Katsaros, F.K. Metal–Carboxylate Interactions in Metal–Alginate Complexes Studied with FTIR Spectroscopy. *Carbohydr. Res.* **2010**, *345*, 469–473, doi:https://doi.org/10.1016/j.carres.2009.12.010.

3. Hua, S.; Ma, H.; Li, X.; Yang, H.; Wang, A. PH-Sensitive Sodium Alginate/Poly(Vinyl Alcohol) Hydrogel Beads Prepared by Combined Ca2+ Crosslinking and Freeze-Thawing Cycles for Controlled Release of Diclofenac Sodium. *Int. J. Biol. Macromol.* **2010**, *46*, 517–523, doi:https://doi.org/10.1016/j.ijbiomac.2010.03.004.

4. Tamasi, G.; Pardini, A.; Croce, R.; Consumi, M.; Leone, G.; Bonechi, C.; Rossi, C.; Magnani, A. Combined Experimental and Multivariate Model Approaches for Glycoalkaloid Quantification in Tomatoes. *Molecules* 2021, *26*.

5. Grasel, F. dos S.; Ferrão, M.F.; Wolf, C.R. Development of Methodology for Identification the Nature of the Polyphenolic Extracts by FTIR Associated with Multivariate Analysis. *Spectrochim. Acta Part A Mol. Biomol. Spectrosc.* **2016**, *153*, 94–101, doi:https://doi.org/10.1016/j.saa.2015.08.020.

6. Virág, D.; Naár, Z.; Kiss, A. Microbial Toxicity of Pesticide Derivatives Produced with UV-Photodegradation. *Bull. Environ. Contam. Toxicol.* **2007**, *79*, 356–359, doi:10.1007/s00128-007-9230-7.

7. Gallagher, L.; Smith, A.; Kavanagh, K.; Devereux, M.; Colleran, J.; Breslin, C.; Richards, K.G.; McCann, M.; Rooney, A.D. Preparation and Antimicrobial Properties of Alginate and Serum Albumin/Glutaraldehyde Hydrogels Impregnated with Silver(I) Ions. *Chemistry (Easton).* 2021, *3*, 672–686.
